# Bridging the Gap from Molecular Surveillance to Programmatic Decisions for Malaria Control and Elimination

**DOI:** 10.4269/ajtmh.22-0749

**Published:** 2023-12-26

**Authors:** Monica Golumbeanu, Constant A. V. Edi, Manuel W. Hetzel, Cristian Koepfli, Christian Nsanzabana

**Affiliations:** ^1^ Swiss Tropical and Public Health Institute, Allschwil, Switzerland;; ^2^ University of Basel, Basel, Switzerland;; ^3^ Centre Suisse de Recherches Scientifiques, Abidjan, Cote d’Ivoire;; ^4^ Department of Biological Sciences and Eck Institute of Global Health, University of Notre Dame, Notre Dame, Indiana

## Abstract

An increasing number of molecular and genomic assays are available to study malaria parasite populations. However, so far they have played a marginal role in informing policy and programmatic decision-making. Currently, molecular data are mainly used for monitoring drug efficacy against *Plasmodium falciparum*; assessing molecular markers of drug and insecticide resistance; and assessing *P. falciparum* histidine-rich protein 2 and 3 genes (*Pfhrp2/3*) deletion. We argue that additional use cases for molecular routine surveillance could be implemented in the near future, especially in transmission settings approaching elimination. These would include using quantitative polymerase chain reaction to monitor the prevalence of sub-patent infections in asymptomatic carriers, monitoring parasite genetic diversity as transmission intensity is changing, using genomic data to determine the origin of imported infections and characterize transmission chains in settings with very low malaria transmission, and using serology to monitor recent and past exposures in low-transmission settings. Molecular surveillance could inform control programs on adapting novel strategies, such as reactive case detection or focal mass drug administration, and help evaluate the impact of interventions currently in place. To better integrate molecular and genomic data into control program decision-making, engagement of national malaria control experts is crucial. Local laboratory capacity needs to be strengthened, shortening the time from sample collection to data availability. Here, we discuss opportunities and challenges of the use of molecular and genomic data for supporting malaria control and elimination efforts, as well as the avenues to link molecular and genomic data with gold standard epidemiological measurements through mathematical modeling.

## INTRODUCTION

Increased coverage with effective malaria control interventions has led to a decrease in the transmission of *Plasmodium* parasites and a notable reduction in the malaria burden across large parts of the malaria-endemic world.^1^^,^^2^ However, increasing subnational heterogeneity in malaria transmission as a consequence of varying uptake and the effectiveness of control measures requires more detailed and granular data for targeting and tailoring intervention packages. As countries scale up their malaria control efforts and aim at eventually achieving elimination, transforming malaria surveillance into a core intervention becomes increasingly important.^3^ In practice, surveillance data need to play an increasing role in programmatic decision-making and day-to-day response action.

Several initiatives are underway to improve the collection and reporting of routine surveillance data, most notably the large-scale rollout of standardized, country-owned, and locally customizable systems such as DHIS 2 (formerly District Health Information Software).^4^ Extensions to DHIS2 and other locally developed systems increasingly allow programs to move from aggregate reporting of clinical cases to individual case–based reporting that forms the basis for case investigation and classification, a requirement for eventual certification of elimination.^5^

To date, efforts toward consolidating and scaling up routine surveillance for the purpose of programmatic decision-making have focused mainly on malariological indicators that are based on well-established diagnostic tools such as microscopy, a rapid diagnostic test (RDT), or morphological identification of mosquitoes.^6^^–^^8^ Although crucial for monitoring malaria transmission and burden, these data capture only part of the complex malaria transmission dynamics in a given geographical region.^9^ Furthermore, not all of these indicators can be collected in all settings.

As parasite and mosquito genetic pools are continuously shaped by control strategies, genomic data provide a complementary tool to understand underlying transmission in response to the applied interventions.^10^ Molecular diagnostic tools can provide a more accurate picture of ongoing transmission,^11^ and they may hence have the potential to improve malaria surveillance efforts. However, to date, molecular and genomic data have been rarely used in routine surveillance^12^^,^^13^ and are often conducted by research institutions that lack programmatic perspective and decision-making power. Their use is limited, for example, by a lack of adequate infrastructure and well-trained staff in many malaria-endemic countries, especially at the National Malaria Control Program (NMCP) level. Moreover, for some molecular techniques, there is a lack of sufficient evidence and hands-on guidance for their programmatic implementation and application.^14^

In this review paper, we present current use cases of molecular techniques to guide malaria strategies and explore future use cases of molecular surveillance to inform programmatic decisions for malaria control and elimination efforts.

## CURRENT USE CASES OF MOLECULAR TECHNIQUES TO INFORM MALARIA PROGRAMMATIC DECISIONS

There are several use cases for malaria molecular monitoring, but only four of them are currently recommended for routine surveillance, including the distinction between recrudescence and new infections in therapeutic efficacy studies (TESs) for *Plasmodium falciparum*^15^ and monitoring of insecticide resistance.^16^ Although guidelines also exist for monitoring *P. falciparum* histidine-rich protein 2 and 3 (*Pfhrp2/3*) genes deletion^17^ and molecular markers of resistance,^18^^,^^19^ they are rarely systematically performed as part of routine surveillance.

In the following section, we provide an overview of the current and prospective use cases of molecular tools for guiding programmatic decisions, also summarized in Table 1.

**Table 1:** Summary of current and prospective use cases of molecular tools for guiding programmatic decisions

Use Case	Description (molecular techniques)	Strengths and Weaknesses
**Current use cases**		
Therapeutic efficacy studies	Use highly polymorphic markers to differentiate recrudescence from new infection (nested PCR followed by gel or capillary electrophoresis, PCR-RFLP, NGS)	+ Drug efficacy estimates comparable between areas of different transmission intensity − Follow-up of patients is required − PCR limitation in detecting minority clones − Reproducibility between laboratories
*Pfhrp2/Pfhrp3* deletion typing (diagnostic resistance monitoring)	Uses nested PCR, qPCR, or digital PCR, Sanger sequencing, NGS	+ Yields conclusive data on deletion status, as false-negative *Pfhrp2*-based RDTs can have other causes − Nondetection of deleted parasites in polyclonal infections
Drug resistance monitoring	Uses established genetic markers to assess the frequency of genotypes associated with drug resistance (nested PCR-RFLP, qPCR, Sanger sequencing, NGS)	+ Characterizes resistance emergence and spread + No follow-up of patient needed; can be used on archived blood samples − Does not provide a direct estimate of treatment efficacy
Insecticide resistance monitoring	Uses established genetic markers to assess the frequency of genotypes associated with insecticide resistance (nested PCR, PCR-RFLP, qPCR, Sanger sequencing, NGS)	+ Characterizes resistance emergence and spread + Affordable and scalable compared with phenotypic studies − Cannot provide a direct relationship with insecticide efficacy
**Prospective use cases**		
Sero-surveillance	Uses established antibodies to measure changes in transmission intensity (ELISA, bead-based assays, protein microarrays)	+ Allows for a better understanding of transmission heterogeneity in low-transmission settings − Acquired immunity may be a major confounder
Detecting the sub-patent and asymptomatic reservoir	Uses highly sensitive diagnostic methods to screen for low-density, sub-patent infections (nested PCR and gel electrophoresis, LAMP, qPCR)	+ Provides an accurate view of the transmission reservoir and where to target interventions + Increasingly available in reference laboratories in endemic countries − Currently not scalable for point of care − Additional benefit over other tools (case numbers, prevalence by mRDT) unclear − Sensitivity depending on the target gene (*18 s*, *varATS*, etc.) and the matrix used for sample collection (whole blood vs. dried blood spot)
Genomic measures as surrogate markers for transmission changes	Use nPCR/gel-based genotyping, PCR–capillary electrophoresis, qPCR (HRM), NGS	+ May be more scalable and affordable than other measures of transmission intensity, especially in areas with low malaria transmission − Links between transmission intensity and parasite diversity/multiplicity are complex and not well understood
Characterizing transmission chains	Identifies hot spots of transmission and sources of importation; tracks cases in the event of an outbreak (NPCR/gel-based genotyping, PCR–capillary electrophoresis, qPCR [HRM], NGS)	+ May allow for new strategies to prevent importation/break transmission chains − Added value compared with travel history reporting by patients not clear

+ = strengths; − = weaknesses; ELISA = enzyme-linked immunosorbent assay; HRM = high-resolution melting; LAMP = loop-mediated isothermal amplification; mRDT = malaria rapid diagnostic testing; NGS = next generation sequencing; nPCR = nested polymerase chain reaction; PCR = polymerase chain reaction; PCR-RFLP = polymerase chain reaction–restriction fragment length polymorphism; qPCR = quantitative polymerase chain reaction.

### Therapeutic efficacy studies.

Polymerase chain reaction (PCR) correction (i.e., comparing the genotype of parasites collected from a patient at enrollment with the genotype of parasites found in the same patient during follow-up) allows distinguishing a new infection (newly acquired genotypes) from a recrudescence (same genotype[s]) in *P. falciparum* infections. Highly diverse–size polymorphic markers, such as *P. falciparum* merozoite surface protein 1 and 2 (*Pfmsp1* and *Pfmsp2*) and microsatellites, are used to differentiate recrudescence from new infection by comparing *P. falciparum* genotypes in pretreatment samples and any posttreatment sample with parasites detectable by microscopy or PCR.^20^^,^^21^ The sensitivity of the different markers in detecting minority clones in polyclonal infections is the main limitation of the techniques used to differentiate recrudescence and new infection.^22^^,^^23^ The recommended WHO protocol for molecular monitoring during clinical trials was recently amended to consider this limitation. Accordingly, the glutamate-rich protein gene (*Pfglurp*) marker was replaced with microsatellite markers owing to the limitation of *Pfglurp* in detecting minority clones in polyclonal infections.^21^ Nevertheless, sampling at a single time point can also result in missed clones because of parasite sequestration and fluctuation in parasite densities.^24^^,^^25^ Furthermore, different data analysis algorithms have been proposed,^26^^–^^29^ and a consensus still needs to emerge about how to validate them.

Polymerase chain reaction correction is still often performed using PCR and agarose gel–based assays. These assays have lower discriminatory power in distinguishing recrudescence from new infection and provide inaccurate estimates of PCR-corrected drug efficacy. There is a need to implement techniques with improved discriminatory power based on capillary electrophoresis or amplicon deep sequencing to precisely estimate amplicon fragment sizes and precisely call haplotypes, respectively; however, many challenges are hampering their wide adoption. Although amplicon deep sequencing (i.e., Illumina sequencing of highly diverse amplicons of a few hundred base pairs) provides higher sensitivity^30^ and results in higher detectability of minority clones,^31^ no guidelines currently exist for assay validation, minimum criteria of assay acceptance, and data reporting. This makes it challenging to compare data over time or between different laboratories. Moreover, the costs associated with implementation of this technology, challenges in reagents procurement, training, and limited capacity in data analysis are still major obstacles for implementation in malaria-endemic countries.

The current system based on TESs, conducted in a few sentinel sites, has many limitations. First, because of logistical and economic constraints, TESs are conducted at best every 2 years, although many countries still fail to reach that goal. Second, in high-transmission settings, delayed parasite clearance, which is the hallmark of artemisinin partial resistance, is confounded by a high level of acquired immunity, making it a poor indicator of the emergence of artemisinin partial resistance in these settings.^32^ Third, TESs are challenging to conduct in low-transmission settings owing to the low number of malaria cases. Additional molecular data, such as the prevalence or frequency of drug resistance markers, could offer a complementary or alternative approach to monitor antimalarial drug resistance, inform the best locations to conduct TES, and confirm resistance.^33^

### Markers of drug resistance.

The molecular basis of resistance to many antimalarials is well understood; thus, typing of respective loci can provide information on the prevalence or frequency of genotypes associated with drug resistance. These results are not confounded by patient immunity and may help to detect early emergence of drug resistance.^34^ For artemisinin partial resistance, mutations in the *PfKelch13* gene have been associated with delayed parasite clearance after treatment with artemisinin combination therapies (ACTs)^35^^,^^36^ or artemisinin monotherapy.^37^^,^^38^ Detection of *kelch13* mutations has proven highly effective in monitoring the spread of resistance across the Greater Mekong subregion.^39^^,^^40^ In contrast to TESs, which require follow-up of patients at multiple time points, monitoring resistance markers can be conducted on any blood sample collected from clinical patients or asymptomatic carriers. It is an affordable and highly scalable strategy. Nevertheless, well-equipped laboratories with well-trained staff are required to conduct the analyses using the most appropriate techniques.^41^

Not all infections with a drug-resistant parasite will result in treatment failure, as resistant infections may be cleared in semi-immune individuals. As a result, there is not always a direct relationship between molecular markers of resistance and treatment failure.^42^ However, increasing prevalence or frequency of validated molecular markers has often been associated with increasing treatment failure,^43^^–^^46^ and large pooled analyses have confirmed the selection of specific resistance markers after treatment with most currently used ACTs (i.e., artemether-lumefantrine and artesunate-amodiaquine).^47^^,^^48^

Most current drug resistance–marker surveillance activities focus on a few geographical sites and are conducted irregularly. Repeated studies are needed to monitor molecular markers and their dynamics over time and space. To better guide policymakers in antimalarial treatment policy change, the development of tools to dynamically map the prevalence of resistance markers and predict treatment outcomes on a population level is needed.^49^^,^^50^

### Diagnostic resistance.

Malaria case management is based on prompt diagnosis and treatment of confirmed cases. This strategy relies on easy access to malaria diagnosis at each level of the health system. Over the last 10 years, malaria RDTs (mRDTs) have become the mainstay for malaria diagnosis in malaria-endemic countries, especially in sub-Saharan Africa.^2^^,^^51^ However, this strategy is threatened by the emergence of parasites lacking the *Pfhrp2* and *3* genes, the target of the most sensitive available RDTs for *P. falciparum*.^52^^–^^54^

*Plasmodium falciparum* parasites lacking the *Pfhrp2/3* genes were initially reported from Peru^55^ and since then in many other malaria-endemic countries, especially in the Horn of Africa.^56^^,^^57^ Two countries in sub-Saharan Africa (i.e., Eritrea and Ethiopia) have reported a very high prevalence of *Pfhrp2/3* genes deletion, leading Eritrea to change its national malaria diagnosis policy from histidine-rich protein 2 (HRP2)-based RDTs to plasmodium lactate dehydrogenase (pLDH)-based RDTs.^58^^–^^60^

The WHO has developed guidance for monitoring *Pfhrp2/3* deletions,^17^^,^^61^ and multiple molecular assays for deletion typing are available.^62^^–^^65^ There is a need to develop laboratory capacity for applying these assays and for the associated high throughput molecular analyses in malaria-endemic countries. Such a laboratory is currently being established in Ethiopia (C. K., personal communication).

### Insecticide resistance.

Susceptibility or intensity bioassays, including the CDC’s bottle bioassays, the World Health Organization Pesticide Evaluation Scheme cone bioassay, and the WHO bottle bioassay, remain the gold standard for monitoring vector resistance to insecticides,^66^ but logistical challenges and high intra-assay variability impede surveillance.^67^ Molecular assays are currently performed only to confirm the underlying mechanism of resistance after detection of phenotypic resistance with bioassays.^68^ However, despite previous studies showing functional links between molecular markers and insecticide resistance,^69^ bioassay outcomes do not always correlate with molecular data.^70^ Although the genotype–phenotype relationship for insecticide resistance is not fully understood, bioassay data may be additionally impacted by other factors, such as environmental factors or metabolic resistance,^67^^,^^71^ impeding their association with molecular data. It is currently recommended that molecular species characterization be conducted after the bioassays to confirm morphological characterization and to identify species groups that cannot be differentiated morphologically.^16^ Logistical challenges, high intra-assay variability, and data interpretation and standardization challenges associated with bioassays impede insecticide resistance surveillance.^72^^,^^73^

Several molecular markers for resistance to each major insecticide class have been established. One such example is knockdown resistance (*kdr*) mutations conferring resistance to dichlorodiphenyltrichloroethane and pyrethroids.^74^ Molecular surveillance can be used to monitor temporal and spatial trends of vector resistance, although this strategy is not yet commonly used.^75^^,^^76^ Despite implementation of monitoring of markers such as *kdr* mutations in several locations,^77^^–^^80^ little is known about the correlation between the prevalence of molecular markers and insecticide efficacy in the field, and more data are needed to fill this gap.^81^ Understanding of the current status of insecticide resistance and the drivers of vector control efficacy can be addressed through molecular surveillance by simultaneously collecting bioassay data, field efficacy of insecticide-based interventions, and the prevalence of molecular markers.

## PROSPECTIVE USE CASES FOR MOLECULAR TOOLS

### Detection of the malaria transmission reservoir.

Currently, the commonly used mRDTs are missing a significant number of infections both in low- and high-transmission settings, especially low-density infections and parasites with deletion of *Pfhrp2/3* genes.^82^^–^^84^ Polymerase chain reaction (or quantitative PCR [qPCR]) is substantially more sensitive than microscopy or an RDT, reaching a limit of detection of < 1 parasite/µL of blood. Polymerase chain reaction screening can be applied to determine the proportion of infections missed by routine diagnosis and to screen for low-density, sub-patent individuals to identify hidden parasite reservoirs. Numerous studies have shown a large sub-patent reservoir under all transmission intensities.^85^^,^^86^ Although PCR was often only available in a relatively small number of research laboratories in the past, slowing down time to results, it is now widely available in endemic countries. Furthermore, portable devices allow screening on-site and thus a much faster turnaround time.^87^^–^^89^

Despite its wide use in research, PCR-based molecular screening for infections has led directly to interventions or policy change in only a few documented cases. In Myanmar, qPCR is usually applied to stratify villages into low or high prevalence, with the latter receiving mass drug administration (MDA).^90^ However, although estimates by qPCR are highly accurate, the cost is high, and screening by an RDT combined with a lower detection prevalence threshold for deploying MDA might be more cost-effective. Another example is the Chinese 1-3-7 approach in which confirmation of infection by qPCR is established before case investigation is conducted.^91^ According to this approach, cases are reported within 1 day and investigated within 3 days, and reactive investigation of the focus is conducted within 7 days. Polymerase chain reaction confirmation is warranted owing to the relatively small number of cases and extensive investigation of each case. On the opposite, during the elimination phase in Sri Lanka, microscopy was the main method used for malaria diagnosis, and RDTs results were always confirmed by microscopy. However, this required the establishment of an extensive microscopy quality assurance program, including proficiency testing and continuous training of the microscopists.^92^

Molecular tools such as qPCR might become more widely integrated into routine surveillance, especially in low-transmission areas or in settings advancing toward elimination.

Polymerase chain reaction has also been established in reference laboratories in other countries that are in the elimination phase (e.g., Afghanistan).^93^ Furthermore, it has been frequently used in research studies to assess the impact of interventions^94^ and for monitoring sub-patent infections.^95^^,^^96^ To increase throughput and reduce costs, multiple samples can be pooled for qPCR analysis. If a pool is positive, samples can be tested individually.^97^^,^^98^ It remains to be seen whether the extensive infrastructure for molecular testing built up in many countries in response to COVID-19 may in the future be leveraged for the molecular surveillance of other pathogens, including malaria.^99^

### Multiplicity of infection (MOI) as a proxy for transmission intensity.

Different indices of genetic diversity are being evaluated as surrogate markers for transmission intensity. The MOI (or complexity of infection) is among the most frequently reported genetic measures. It can be easily determined using any of multiple genotyping techniques. Some small-scale, geographic-specific studies have found a strong correlation between MOI and other measures of transmission intensity (e.g., clinical incidence or prevalence), in particular over extended periods of time. Nevertheless, a systematic review of the relationship between MOI and prevalence did not find a clear pattern.^100^ Likewise, the relationship between other genetic diversity metrics and transmission intensity is poorly characterized. It appears that MOI starts to decline only once transmission has been reduced to very low levels (reviewed in Koepfli and Mueller^101^ and Noviyanti et al.^102^). Owing to these complexities, genomic indicators are not routinely used by control programs.

### Parasite relatedness, detecting importation, and characterizing transmission chains.

More sophisticated genomic analysis, including genotyping a higher number of markers or whole genome sequencing (WGS), and advanced tools for data analysis can provide insights into parasite movements across space and time.^103^ Once transmission is very low, it becomes crucial to differentiate between imported and locally transmitted cases. Parasite genotyping might be able to do that, but it will rely on a good understanding of the genomic composition of the local parasite population as well as populations that might serve as sources for importation.^101^^,^^104^ Baseline and subsequent cross-sectional surveys to generate WGS data in low- to very low–transmission settings would provide a better understanding in this regard,^10^ as they can be used in mathematical models to elucidate the structure of parasite populations and transmission networks to target and tailor interventions accordingly.^105^^–^^107^ Almost all existing studies on genetic diversity, MOI, and transmission networks were conducted at a time when changes in transmission were already evident by changing clinical case numbers. Likewise, self-reported travel histories used to be sufficient to classify a case as imported versus locally transmitted. However, self-reported travels may not be able to reliably disentangle local transmission chains, as transmission in areas approaching elimination is very complex and may be due mainly to imported cases, despite a large local sub-patent parasite reservoir.^108^ Moreover, compared with travel history and mobile phone data, genomic data can provide better estimates of parasite movements and connectivity over longer distances,^109^ paving the way to regional activities for malaria control and elimination.

### Sero-surveillance.

Serological markers to monitor recent or past exposure to parasites or vectors hold great promise for monitoring control efforts and may serve to identify at-risk populations or hot spots, where preventive measures should be implemented. Applications have been reviewed in detail elsewhere^110^^,^^111^ and are summarized briefly here.

In particular, when transmission becomes very low, clinical cases or asymptomatic infections are too few to provide meaningful information in heterogeneity of transmission or recent changes in response to control. Measuring antibody titers as markers of past exposure to parasites can provide higher power to assess differences. For example, in western Kenya, seroprevalence across multiple clusters was around 5-fold higher than prevalence by PCR and 10-fold higher than prevalence by RDT.^112^ Seroprevalence data thus allowed for a better understanding of heterogeneity in transmission than parasite prevalence data alone.

When very few parasites are present in a population, sero-surveillance can be applied to quantify transmission in the population. With use of different antibodies with different half-lives, it may be possible to assess cumulative and recent exposures. Recent studies have shown that antibodies such as *Ama-1* and *msp1* may be associated with cumulative exposure, whereas *Etramp* and *Glurp* are associated with recent or current infection.^113^^,^^114^ Even in the absence of current or recent infections, individuals at higher exposure to vectors are expected to be at risk of getting infected. Bites of infected and uninfected mosquitoes result in a relatively short-lived immune response. The gSG6-P1 peptide was identified as a marker to *Anopheles* bites^115^ and was used to stratify exposure in multiple settings.^116^^,^^117^ Antibodies for this assay were successfully extracted from archived RDTs collected by the National Malaria Elimination Program in Bangladesh,^118^ thus minimizing the efforts for field collection of specimens. Highly multiplexed assays have also been developed allowing assessment of hundreds or thousands of antibodies at the same time and helping to identify new markers for parasite or mosquito exposure.^119^^,^^120^

The main limitation of all these molecular techniques is the requirement of human blood samples. Because it may not always be possible to collect the required samples for molecular analyses, an alternative would be to use mosquitoes. Indeed, DNA extracted from mosquitoes may be suitable not only for insecticide resistance monitoring but also for parasite drug resistance monitoring or *Pfhrp2/3* deletion typing. Mosquitoes can thus also be used as a source of parasite DNA, and previous studies have shown that this could be a fast and cost-effective strategy,^121^^,^^122^ alleviating the need to collect blood samples from humans. However, this strategy will be likely applied in high-transmission settings, where routine entomological surveillance is regularly conducted and mosquito infection rates are assessed.

## THE ROLE OF MATHEMATICAL MODELING

Data derived from routine epidemiological surveillance and representative surveys have been previously successfully used to guide policy decisions in numerous use cases.^123^^–^^125^ For this purpose, mathematical modeling and statistical analyses have proven instrumental in extracting quantitative evidence from collected surveillance data and informing programmatic decisions. Frameworks using epidemiological data for statistical analyses and to parameterize mathematical models of malaria transmission have been set up and used by countries to guide their national malaria strategic plans.^126^ A nonexhaustive list of these applications includes identification and stratification of malaria risk,^127^^–^^129^ optimization of interventions for national strategic planning,^130^ and support for funding requests to the Global Fund.^131^^,^^132^ Furthermore, routine surveillance data and quantitative risk assessment combined with statistical and model-based analyses have constituted a key component of the National Malaria Elimination Program in China.^133^

Despite the existing data and modeling frameworks for decision-making and their uptake by certain NMCPs, only a few small-scale applications have included molecular surveillance data. Of note, in this context, mathematical models have been previously used to link parasite diversity to changes in transmission intensity,^134^^,^^135^ map the drivers and spread of *Pfhrp2* deletions,^136^ and drug resistance,^137^^,^^138^ identify outbreaks,^139^ and characterize flows of infections.^107^ However, despite their usefulness,^104^ these applications have been mostly restricted to a specific geographical setting rather than applied countrywide and were performed mostly for research purposes rather than with an actionable programmatic implication for malaria control and elimination strategies. The development of analytical methods and extension of current mathematical models to include molecular surveillance data could bring valuable insights and complement the current efforts of national malaria control and elimination programs.

Mathematical modeling can be used to leverage the available molecular data collected so far. In the context of TESs, the current algorithm recommended by the WHO to distinguish recrudescence from new infections has some limitations, as it does not consider uncertainty associated with the markers and the technique used for genotyping.^21^^,^^140^ More robust algorithms providing a certain degree of uncertainty in genotyping results are needed to help policymakers make the most appropriate decisions. Several recent algorithms have been developed for distinguishing recrudescence from new infections based on dynamical modeling and Bayesian statistics.^26^^–^^28^ A systematic benchmarking of these methods on multiple existing molecular datasets would allow establishing a standardized algorithm that could be readily applicable in the countries where TESs are conducted.

Having been previously used for estimating the temporal and geographic distribution of malaria burden,^129^^,^^141^^,^^142^ geospatial methods can be used to construct monitoring tools for the spread of *Pfhrp2* deletions and drug resistance markers.^49^^,^^50^ These tools could be incorporated in existing monitoring dashboards (e.g., DHIS2) that are already in place, but are currently based mostly on epidemiological indicators. However, although informative, the spatiotemporal distribution of molecular markers is challenging to interpret for programmatic purposes. For instance, the relationship between molecular markers of drug or insecticide resistance and drug efficacy or insecticide resistance, respectively, is yet unclear as many confounding factors play an important role.^143^^–^^145^ Previous modeling analyses have explored the potential contribution of biological, epidemiological, and treatment factors of the establishment and spread of drug resistance,^137^^,^^146^ as well as optimal treatment schemes to prevent drug resistance establishment and spread.^147^^,^^148^ Nevertheless, there remains a need to develop complementary tools to quantitatively understand the relationship between the prevalence of molecular markers of drug resistance and treatment efficacy by leveraging the collected molecular marker data. By analysis of existing datasets, quantitative methods can be developed to further understand these relationships. Developing models that can estimate these relationships on a population level and over time could provide a valuable tool to estimate the efficacy life span of deployed antimalarial drugs or insecticides and help policymakers plan well ahead of potential changes in drug or insecticide policy.

## INCORPORATING MOLECULAR SURVEILLANCE DATA INTO PROGRAMMATIC AND POLICY DECISION-MAKING

Combining molecular surveillance data with existing routine epidemiological indicators to guide NMCPs comes with its own challenges. First, collection of molecular data is not yet standardized, and countries need capacity for sample collection, storage, processing, and analysis. Although sample size recommendations are available for some use cases (e.g., for *Pfhrp2/3* deletion genotyping), no guidance exists on the number of samples to be screened to determine the size of the sub-patent reservoir or the required sensitivity of the PCR assay.

Furthermore, currently there is no gold standard for data collection, interpretation, and integration with current standard epidemiological approaches. Specifically, as most of the routine epidemiological indicators are reported on a monthly basis, it is not clear whether the molecular indicators would need to be collected at the same resolution, as well as the number of samples needed to calculate them. In this case, modeling can guide the design of sample collection under different assumptions about parasite and human populations and determine at what level a policy change is needed.^149^ Furthermore, the definition of molecular indicators is not yet established as opposed to the standard epidemiological indicators. So far, complexity of infection and other measures derived from population genetics, such as heterozygosity and identity by descent, have been used to define parasite population diversity, but it is unclear how they relate to the epidemiological indicators in space and time.

To date, molecular studies are often conducted separately from other surveillance efforts and lack systematic sample collection and statistical power. Data and interpretation are reported as peer-reviewed manuscripts, often years after sample collection. For an integrated molecular surveillance program, systematic sampling and molecular analysis are recommended, as well as fast communication of results to policymakers. For example, baseline samples collected for a TES could be screened by different RDTs per WHO recommendation and the same samples typed for *Pfhrp2/3* deletion. Molecular prevalence surveys could be linked to data from health centers to understand whether the number of clinical cases diagnosed by microscopy or RDT can serve as a surrogate marker to identify the spatial or temporal heterogeneity of subclinical and sub-patent infections. Specifically, these data have been included in analyses to predict the reservoir of submicroscopic infections in the community using Bayesian statistical models or log-linear regression models as previously described.^150^^–^^152^ These approaches could support designing targeted interventions at the community level using incidence data from routine malaria surveillance. Finally, optimal sampling strategies need to be developed within the framework of routine surveillance, and reporting and visualization tools must be developed to facilitate data access and use for policymakers.^33^

Several efforts are currently aimed at developing molecular surveillance systems capable of generating relevant evidence for guiding programmatic decisions. A preeminent example is a large-scale study being conducted in Mozambique during 2022–2023.^153^ Accordingly, a protocol is being established and implemented for three use cases, namely monitoring drug and diagnostic resistance, identifying sources of transmission, and evaluating transmission levels and the impact of interventions. Data are being collected across a wide range of malaria transmission settings, ranging from low to high levels of transmission and exploring different sampling approaches. Downstream data analyses and modeling using the data generated in the study include descriptive analyses of observed molecular data trends as well as more elaborate modeling analyses, including stratification of malaria risk and assessment of intervention impact. Several similar efforts are currently ongoing in other sub-Saharan countries such as Tanzania.^154^^,^^155^ Such longitudinal studies provide valuable insights for further understanding the role of molecular surveillance and its complementarity to existing routine epidemiological surveillance in guiding malaria control programs.

**Figure 1.**
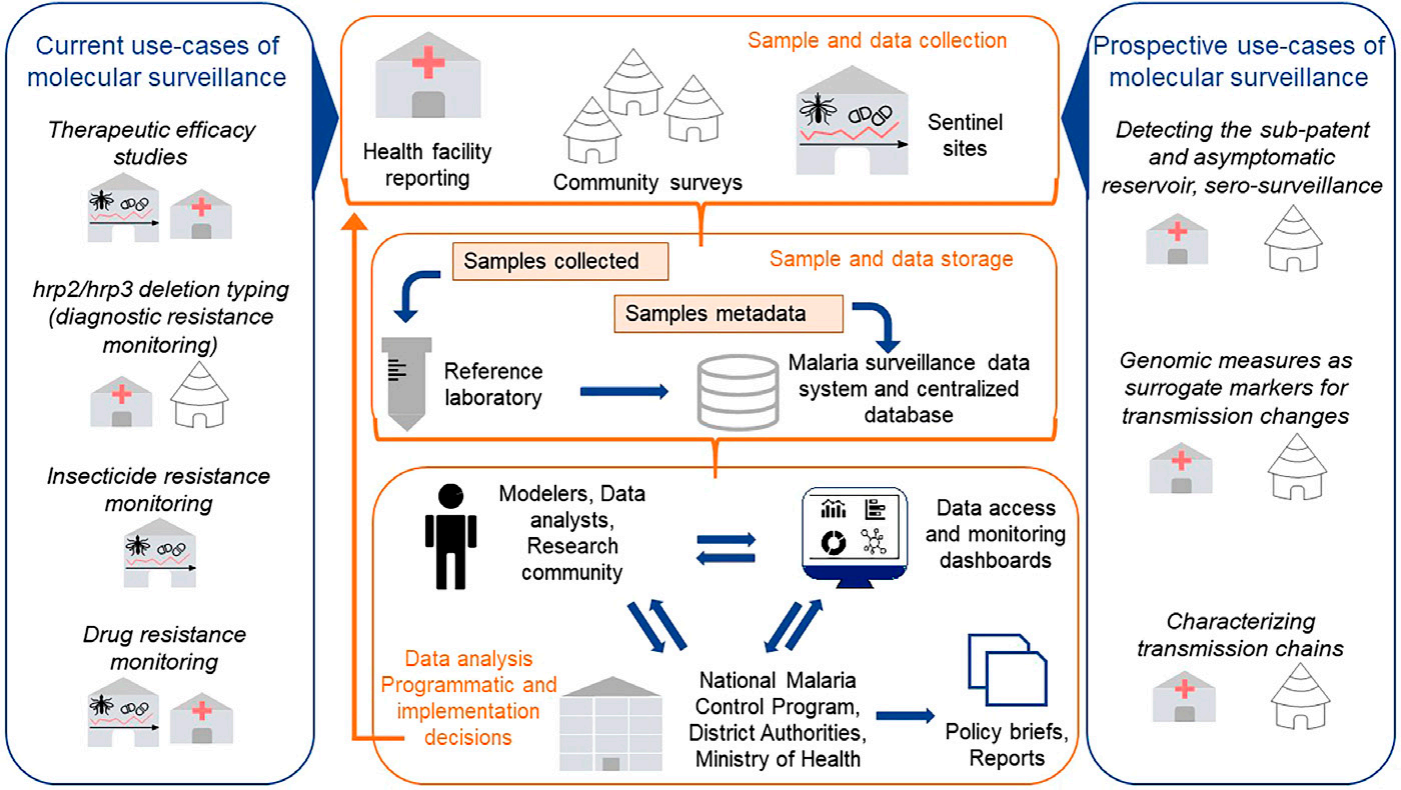
Current and prospective use cases of molecular surveillance and their potential integration within malaria surveillance systems. Samples and sample metadata are collected at the community level through cross-sectional surveys, at a health facility, and at sentinel sites. Anonymized samples are shipped to the reference laboratory or any laboratory performing the analysis, whereas the metadata are sent to a centralized database. Once the samples have been analyzed, the results are sent to the central malaria database and linked to the metadata. Data analysts and modelers will use this central database to analyze and model the data, producing key malaria metrics that will be accessible through surveillance dashboards to key malaria stakeholders at the national (ministry of health, National Malaria Control Program) and regional (district health authorities) levels. These data will be used by the stakeholders to target their interventions and produce policy briefs and surveillance reports.

To facilitate incorporating molecular surveillance data into programmatic and policy decision-making, the different use cases presented here would first need to be adapted to and incorporated in routine surveillance systems. We argue that this would entail a modular organization of processes consisting of sample and data collection, storage and downstream data analysis, and iterative dialogue using generated quantitative evidence and results to guide implementation decisions (Figure 1). Depending on the use case, molecular data collection may be performed at health facilities, through community surveys, and in sentinel sites. The frequency of sampling for the current use cases could follow current WHO guidelines (e.g., every 2 years for TES and drug resistance monitoring, every year for insecticide resistance monitoring), but it could be adjusted depending on the results of data analysis (e.g., more frequent if drug resistance markers are detected). Molecular surveillance for the prospective use cases would be best suited in settings with very low malaria transmission, close to elimination and prior implementation of reactive interventions or in the event of an outbreak. Establishing reference laboratories at the country level as well as standardization of sample collection methods and centralized data storage would facilitate comparison of assays in different locations, easy access to data, and subsequent data analyses.^156^ Finally, a continuous dialogue between data analysts or modelers and official bodies such as the NMCPs, ministries of health, or other authorities would be crucial for leveraging the information extracted from the molecular data to inform programmatic and implementation decisions.

## THE ROLE OF RECENT LABORATORY INFRASTRUCTURE DEVELOPMENT

The COVID-19 pandemic has seen unprecedented, concerted efforts from the global health community in scaling up genomic surveillance in low- and middle-income countries, especially in sub-Saharan Africa. The establishment of the Africa Centers for Disease Control and Prevention (Africa CDC) Institute of Pathogen Genomics, through the Africa Pathogen Genomics Initiative, is a great example.^157^ Indeed, the Africa CDC and the African Society for Laboratory Medicine have partnered with public and private institutions to improve the capacity for genomic surveillance on the continent through continental and regional laboratory hubs. This has dramatically improved the equipment and reagent procurement processes, as well as equipment maintenance, by centralizing the processes. Training activities are coordinated through a few centers of excellence on the continent, providing training and external quality assurance (EQA) schemes. The malaria community needs to build on this momentum to improve molecular surveillance by scaling up activities for recommended routine surveillance use cases and providing evidence about new use cases to define their scope of use and utility in informing public health policies.

As countries are getting access to state-of-the-art techniques for molecular and genomic surveillance, training capacity needs to be increased, and strategies for sustainability and maintaining qualified staff developed. There is a need to develop target product profiles to define the minimum criteria for assay validation and acceptance for the different use cases and to implement adequate EQA schemes to allow laboratories in malaria-endemic countries to implement these new assays.^158^ This will not only allow for data comparison over time and between laboratories and countries but will also greatly improve quality management systems and therefore the quality of data. Improved systems for data collection and collation will be crucial to link the laboratory, clinical, and epidemiological data through data sharing platforms on national and supranational levels—the next step for an improved real-time surveillance system.

## THE WAY FORWARD

The integration of molecular data into routine malaria surveillance has the potential to complement the existing malariological data and substantially improve the quality and accuracy of information generated for decision-making. However, because of the limited funding currently available for malaria control and elimination programs as well as the lack of in-country capacity for implementing the different molecular techniques, cost-effective strategies and use cases for using molecular and genetic surveillance must be developed.

In high-transmission settings, where the reduction of the burden is the first priority, there is a need to improve the quality, accuracy, and reporting of classic epidemiological data (e.g., incidence, prevalence). Three molecular use cases could complement these data: the prevalence of resistance markers for drugs, for vectors, and for *Pfhrp2/3* deletions. However, their use could be optimized to provide more robust data, even though an initially substantial investment would be required.

In low-transmission and elimination settings, where the detection of each malaria infection becomes crucial for elimination programs, the integration of portable PCR devices for reactive case detection could substantially improve the surveillance system. The development of multiplex assays that can detect other pathogens on these devices should be a priority. Indeed, in Southeast Asia, where most countries are moving to elimination, only a very low number of RDTs performed on patients with fever are positive,^159^ making it important for malaria programs to develop a holistic approach and collaborate with other programs. Assays with panels of pathogens specific for each region would improve not only malaria case detection but also prescription practices because in most cases of negative RDT results, health workers tend to give an antibiotic to the patient, potentially impacting antibiotic resistance. Central laboratories would still perform monitoring for drug and vector resistance and *Pfhrp2/3* deletion. In addition, in these settings it will be essential to monitor the impact of the different interventions on the parasite’s genetic diversity, identify sources and sinks of infections, and confirm imported cases and parasite population movements in areas of interest.^160^ Furthermore, serological surveys provide a highly scalable and accessible tool to assess past and recent exposures to parasites and vectors,^113^^,^^114^^,^^161^ allowing stratification of regions with ongoing transmission or where malaria has been eliminated. Indeed, serological assays are cheaper to implement and can be easily used for large-scale analyses. For example, serological data indicating recent exposure either to malaria vectors or parasites in an elimination setting can trigger molecular analysis to confirm local or imported infections, helping the control program to adopt the most appropriate intervention to target local transmission foci or prevent the importation of infections.

With the recent confirmation of partial artemisinin resistance in Rwanda, Uganda, and Eritrea,^162^ there is a need to strengthen the surveillance system for antimalarial drug resistance across the entire sub-Saharan Africa. Consecutive health facility–based cross-sectional surveys across the country could provide valuable information on the spatiotemporal distribution of markers of resistance and potentially inform the selection of the most appropriate sentinel sites for TESs. Therefore, building the capacity for control programs to analyze samples on a regular basis for these cross-sectional surveys is of paramount importance. National reference laboratories could perform the molecular assays or work in collaboration with research laboratories that already have the expertise. The assays performed should not only be limited to molecular markers of resistance but should also include PCR correction to distinguish recrudescence from new infection in TESs, *Pfhrp2/3* deletion, and molecular markers of insecticide resistance. Countries should also leverage the recent capacity developed for pathogen genomic surveillance in malaria-endemic countries through the different initiatives coordinated by the Africa CDC.^163^ Regional centers of excellence established by the Africa CDC could provide training and develop and implement external quality control program schemes. Finally, data analysis and mathematical modeling combining epidemiological and molecular data can provide accurate and robust information through easily accessible monitoring tools to policymakers, upon which they can base their decisions when implementing tailored interventions.

## CONCLUSION

Molecular surveillance can provide essential data for NMCPs, complementing the information provided by classic routine epidemiological indicators, such as case numbers, offering a more in-depth and more granular picture of transmission dynamics in space and time. Key molecular information includes the distribution of drug, diagnostic, and insecticide resistance in all transmission settings, as well as characterization of transmission chains and heterogeneity of transmission in moderate- to low-transmission settings. Although highly valuable, challenges in terms of infrastructure, data management, data analysis including mathematical modeling, and data interpretation need to be addressed in order for molecular and genetic information to be of direct use for decision-making.
